# Metabolomic insights into thiamine-mediated metabolism in treating subclinical ketosis in dairy cows

**DOI:** 10.3389/fvets.2026.1764526

**Published:** 2026-04-13

**Authors:** Fuguang Xue, Fan Zhang, Jintao Xue, Mengjie Sun, Benhai Xiong

**Affiliations:** 1 School of Animal Science and Technology, JiangXi Agricultural University, Nanchang, China; 2 Institute of Animal Science, Chinese Academy of Agricultural Sciences, Beijing, China; 3 Testing Center of Gaotang Market Supervision and Administration, Liaocheng, China

**Keywords:** beta-hydroxybutyrate, dairy cows, subclinical ketosis, succinyl-CoA, thiamine

## Abstract

**Introduction:**

Subclinical ketosis (SCK) is a common condition in dairy cows, primarily caused by the excessive accumulation of *β*-hydroxybutyrate (BHBA). Research has shown that succinyl-CoA is negatively correlated with the production of hepatic BHBA, while it is positively correlated with the utilization of BHBA in peripheral tissues. This correlation is significantly modulated by thiamine levels. Therefore, the purpose of this study is to investigate whether thiamine serves as a critical rate-limiting cofactor in succinyl-CoA metabolism, potentially enhancing BHBA utilization and reducing the incidence of SCK.

**Methods:**

To verify the purpose of the study, 60 SCK dairy cows with comparable body weight, milk yield, and lactation days were selected from a total of 1,126 dairy cows reared in the same environment. The selection was based on the criterion of blood beta-hydroxybutyrate concentration being greater than 1.2 mmol/L. Another 12 healthy dairy cows with similar productive performances were enrolled in the control treatment. Milk yield, milk quality, rumen fermentable parameters, rumen and blood metabolomics were measured to investigate the alleviative effects of thiamine on SCK.

**Results and discussion:**

The results showed that thiamine significantly decreased plasma BHBA concentrations, milk colony-forming units (CFU), and somatic cell counts, while significantly increasing milk yield, milk fat content, acetate levels, and the acetate-to-propionate (A/P) ratio compared to SCK cows (*p* < 0.05). Metabolomic analysis revealed that the upregulated metabolites in both the rumen and blood after thiamine supplementation were significantly enriched in the pathways of pyruvate metabolism, glycolysis or gluconeogenesis, tyrosine metabolism, and glycerophospholipid metabolism, while the downregulated metabolites were mainly enriched in ascorbate and aldarate metabolism, glycerophospholipid metabolism, pentose and glucuronate interconversions, and riboflavin metabolism. Collectively, thiamine supplementation effectively alleviated subclinical ketosis by promoting the mediated catabolism of succinyl-CoA for BHBA utilization, improving ruminal fermentability, and thereby enhancing the milk yield and quality.

## Introduction

Subclinical ketosis (SCK) is primarily caused by an overload of hepatic *β*-oxidation, incomplete catabolism of acetoacetyl-CoA, and consequent accumulation of *β*-hydroxybutyrate (BHBA). This condition is driven by a sustained negative energy balance (NEB) in early-lactation dairy cows (1–3). Global SCK incidence averages approximately 22.7%, with rates of 15–30% in Chinese native dairy cattle, significantly limiting dairy productivity and leading to substantial economic losses (4).

Accumulating evidence indicates that elevated BHBA disrupts normal nutrient metabolism such as the inhibition of fructose-1,6-bisphosphatase (FBP2) activity (3) and a reduction in hepatic gluconeogenesis efficiency by approximately 47% (5). Elevated BHBA also correlated with a decline in ruminal starch-utilizing bacteria and a marked suppression (up to 53%) of amylase activity, thereby compromising feed efficiency and the overall production performance (6, 7). Glucose infusion is commonly administered for symptomatic relief of SCK; however, it fails to address the core defects in the breakdown and utilization of BHBA and frequently results in disease recurrence (3). Therefore, clarification of physiological BHBA metabolism and modulatory points is critical for alleviating SCK.

Previous investigations on BHBA metabolism highlighted succinyl-CoA as a central regulatory node for the significant negative correlation with 3-hydroxy-3-methylglutaryl-CoA synthase (HMGCS)—key enzyme in hepatic BHBA synthesis (8) and a positive correlation with the activity of succinyl-CoA:3-ketoacid CoA transferase (SCOT)—key enzyme involved in peripheral BHBA utilization (9). Thus, boosting physiological succinyl-CoA availability may be pivotal in promoting BHBA catabolism and mitigating SCK.

Thiamine acts as an essential coenzyme in the form of thiamine pyrophosphate (TPP) for the *α*-ketoglutarate dehydrogenase complex (*α*-KGDH) in succinyl-CoA biosynthesis within the tricarboxylic acid (TCA) cycle. Thiamine deficiency inhibits the activity of *α*-ketoglutarate dehydrogenase, potentially reducing succinyl-CoA levels, impairing BHBA utilization, and exacerbating ketosis (10, 11). Conversely, thiamine supplementation has been shown to stimulate succinate-producing ruminal bacteria such as *Succinivibrionaceae, Succiniciasticum*, and *Succinivibrio* (26), attenuate inflammatory responses, and potentially further boost succinyl-CoA generation and aid SCK recovery (12, 13). Therefore, thiamine supplementation in SCK dairy cows was investigated in this study to determine its modulatory effects on BHBA synthesis and catabolism and the potential mechanism for alleviating SCK.

## Materials and methods

### Animal preparation and experimental design

Dairy cows were reared at the Bengbu dairy farm, Modern Farming (Wuhe) Co. Ltd., Anhui Province, China (32.92 N, 117.38 E). The care and procedures were followed according to the Chinese Guidelines for Animal Welfare, which were approved by the Animal Care and Use Committee of Jiangxi Agricultural University, with approval number JXAULL-20241024.

A total of 1,126 dairy cows reared in the same environment were first subjected to blood samples 3 h after morning feeding and repeated for 2 days. Cows suffering from subclinical ketosis (SCK) were selected based on the filtrate standard of blood beta-hydroxybutyrate concentration >1.2 mmol/L. A total of 60 SCK dairy cows with similar body weight, milk yield, and lactation days, along with 12 healthy dairy cows with similar body weight, milk yield, lactation days, and parities, were selected for further investigation of the alleviative effects of thiamine supplementation treatment.

All selected dairy cows were separated into the isolated barn and reared individually in small pens. The barn was divided into 12 blocks, each containing 6 cows (one cow from each treatment) with similar body weight, lactation days, and parities. Each cow was considered a replicate, randomly allocated into pens from each block, and recorded individually for the dry matter intake and milk yield. Diets were formulated according to NRC (27) to meet the energy requirements of Holstein dairy cows yielding 40 kg of milk/day with 3.5% milk fat and 3.0% true protein. The nutritional composition of the ingredients for each treatment is shown in Table 1. Diets were fed twice daily at 06:00 and 18:00, and water was available *ad libitum* throughout the whole experiment. Thiamine was supplemented at the dosage used in our previous study (12, 13), and the gradients were set to 120 mg, 180 mg, 240 mg, and 300 mg thiamine/kg of DMI. Simply stated, the average DMI of enrolled dairy cows in each treatment was calculated based on the data collection in a 10-day-long pretreatment trial, and the usage of thiamine in each treatment was supplemented into the premix and further mixed into the TMR to meet the requirement for thiamine ingestion.

**Table 1:** Ingredients and chemical composition of the feedstuff used for dairy cows.

Items	Percentage (%)
Ingredients	
Corn silage	20.5
Ground Corn	19.7
Cottonseed meal	3.4
Alfalfa hay	10.5
Chinese Wildrye	10.2
Distillers dried grains With Solubles (DDGS)	3.1
Steam-flaked corn	12.5
Soybean meal	12
Beet pulp	4.5
Premix^a^	3
NaCl	0.6
Total	100
Chemical composition	
DM	51.2
NE_mf_ (MJ/kg)^b^	7.13
EE	4.56
CP	18.1
ADF	18.6
NDF	27.6
Starch	30.8
Ca	0.69
P	0.44

a The components contained in the premix are as following: Fe, 1,400 mg; Cu, 1,200 mg; Mn, 2,400 mg; Zn, 5,500 mg; Se, 40 mg; Co, 30 mg; I, 90 mg, VA, 900,000 IU; VD, 700,000 IU; VE, 9,000 IU.

b NE_mf_ = {322LBW^0.75^ + [(2092 + 25.1 × LBW) × ADG/(1–0.3 × ADG)]} × F.

LBW, live body weight; ADG, average daily weight gain; F, correction coefficient.

### Milk yield and milk quality measurement

All experimental cows were milked three times per day at 07:00, 14:00, and 21:00, and the milk yield of each cow was automatically recorded by the rotary milking facilities (9JRP-50P2100, Delaval, Israel). Milk was sampled from each cow during the last three consecutive days for the milk quality-related parameter measurement. Milk protein and milk fat were measured using a near-infrared analyzer (MilkoScanTM 7 RM, Foss Electric, Denmark). Somatic cell count (SCC) was measured using a SCC rapid analyzer (Fossomatic 7/7 DC, FOSS).

### Effects of thiamine supplementation on rumen fermentable parameters

Rumen fluid samples from each cow were collected via the esophageal tubing method and divided into two portions. One aliquot was immediately analyzed for ruminal pH (Testo 206-pH, Testo Instruments International (Shanghai) Co., Ltd., Shanghai, China), volatile fatty acids (VFAs) (GC-2010, Shimadzu, Kyoto, Japan), ammonia-N (NH_3_-N, UV-2600 ultraviolet spectrophotometer, Tianmei Ltd., China), and microbial protein (MCP). The remaining was snap-frozen in liquid nitrogen and stored at −80 °C for metabolomic measurement.

### Rumen fluid and blood metabolomic measurement

Cows in the optimal thiamine supplement treatment, control treatment, and SCK treatment were selected based on their productive performance, milk quality, and fermentable indices, and were chosen for ruminal and blood LC/MS-based metabolomic analysis. Methods used here are the same as those described in our previous study (14). Simply stated, metabolites were extracted and separated using a Thermo UHPLC system equipped with an ACQUITY UPLC HSS T3 column (100 mm × 2.1 mm, 1.8 μm; Waters, USA). Detection was performed on a Thermo Q Exactive HF-X mass spectrometer with electrospray ionization operating in both positive and negative modes. Raw data were processed using Progenesis QI 2.3 (Nonlinear Dynamics, Waters, USA) to perform peak alignment and detection. The resulting data matrix included retention times (RTs), mass-to-charge ratios (*m/z*), and peak intensities, which were used for downstream statistical and pathway enrichment analyses.

### Statistical analysis

Milk yield, milk quality, and rumen fermentation variables were firstly subjected to a normal distribution test using the SAS procedure “proc univariate data=test normal” and subsequently received the one-way ANOVA S-N-K test by SAS (SAS Institute, Inc., Cary, NC, USA). Significance is considered when the *p*-value is < 0.05. Metabolomic analysis was performed using ropls (version1.6.2, http://bioconductor.org/packages/release/bioc/html/ropls.html). All filtered metabolites were used for principal component analysis (PCA) in R software (Version 3.15.3, R Core Team, Vienna, Austria). Significantly differential metabolites were identified through the filtration method of variable importance in the projection (VIP) > 1, fold change>2, and *p* < 0.05. All identified differential metabolites were applied for enrichment and pathway analysis.

## Results

### Effect of thiamine supplementation on the productive performances, milk quality, and fermentable parameters of subclinical ketosis dairy cows

The effects of thiamine supplementation on milk yield and milk quality of SCK dairy cows were investigated, and the results are shown in Table 2. Thiamine supplementation significantly decreased the BHBA content (*p* < 0.05), indicating an effective alleviation on SCK. SCK cows showed a significant decrease in milk yield, milk fat content (*p* < 0.05), a decreasing trend in DMI (0.05 < *p* < 0.10), and a significant increase in the CFU and somatic cells compared with CON cows (*p* < 0.05). Thiamine supplement significantly increased the milk yield and milk fat content, while significantly decreasing the CFU and somatic cells compared with SCK cows (*p* < 0.05). No other significant alterations were observed in the remaining parameters.

**Table 2:** Effects of thiamine supplement on the productive performances of subclinical ketosis dairy cows (*n* = 12).

Items	CON	SCK	120 mg TS	180 mg TS	240 mg TS	300 mg TS	SEM	*p*-value
Milk yield (pre)	44.27	44.26	44.21	43.97	44.16	44.13	1.36	0.674
Milk yield (post)	44.12^a^	40.36^b^	42.17^a,b^	43.59^a^	43.44^a^	43.09^a^	1.78	0.031
DMI (pre)	22.46	22.44	22.47	22.41	22.49	22.48	0.86	0.364
DMI (post)	22.37	21.31	21.66	21.87	21.93	21.86	0.79	0.091
Milk efficiency (pre)	1.97	1.97	1.97	1.96	1.96	1.96	0.03	0.596
Milk efficiency (post)	1.97	1.89	1.95	1.99	1.98	1.97	0.03	0.027
*β*-hydroxybutyrate(mmol/L)	0.86^b^	1.41^a^	1.27^a^	1.07^b^	1.04^b^	0.97^b^	0.13	0.024

CON, control treatment; SCK, subclinical ketosis. SCK = subclinical ketosis with thiamine supplement. SEM, standard error of the mean. “a,b” in the same row means a significant discrepancy between all treatments.

Furthermore, the thiamine supplement on the rumen fermentable parameters of SCK dairy cows, including the VFAs, rumen MCP, and rumen NH_3_-N, are shown in Table 3. Acetate, propionate, and butyrate contents significantly decreased in SCK dairy cows compared with CON (*p* < 0.05). Supplementing with thiamine significantly increased the acetate content compared with SCK cows (*p* < 0.05); however, no significant discrepancies were observed in propionate and butyrate contents. A significant increase in the A/P ratio in SCKT-treated dairy cows was detected compared with CON and SCK treatments (*p* < 0.05). The MCP content, ammonia, and ruminal pH showed no significant alteration among all treated dairy cows. Based on the results above, combined economic considerations, the thiamine supplement at 180 mg/kg DMI was selected for further metabolomic analysis (Table 4).

**Table 3:** Effects of thiamine supplement on the milk qualities of subclinical ketosis dairy cows (*n* = 12).

Items	CON	SCK	120 mg TS	180 mg TS	240 mg TS	300 mg TS	SEM	*p*-value
Milk fat	3.76^a^	3.42^b^	3.51^b^	3.61^a^	3.64^a^	3.67^a^	0.16	0.016
Milk protein	3.44	3.31	3.41	3.42	3.41	3.42	0.16	0.153
Non-fat solid	8.56	8.36	8.37	8.5	8.53	8.57	0.34	0.224
Dry matter content	12.32	11.78	12.07	12.11	12.13	12.17	0.32	0.106
Milk density	1.03	1.029	1.029	1.029	1.029	1.029	0.004	0.801
Acidity	13.1	13.1	13.1	13.1	13.1	13.1	0.04	0.534
CFU (10^3^)	6.32	6.72	6.58	6.61	6.49	6.51	1.34	0.227
Somatic cell (10^3^)	5.21^b^	7.60^a^	6.23^b^	5.46^b^	5.86^b^	5.52^b^	0.46	0.037

CON, control treatment; SCK, subclinical ketosis. SCK = subclinical ketosis with thiamine supplement. SEM, standard error of the mean. “a,b” in the same row means a significant discrepancy between all treatments.

**Table 4:** Effects of thiamine supplement on ruminal fermentability of subclinical ketosis dairy cows (*n* = 12).

Items	CON	SCK	120 mg TS	180 mg TS	240 mg TS	300 mg TS	SEM	*p*-value
Ruminal pH	5.97	6.01	6.03	6.02	5.99	5.98	0.094	0.216
MCP (mg /dL)	24.16^a^	21.23^b^	23.41^a^	23.54^a^	23.47^a^	23.42^a^	0.741	0.043
Acetate (mmol/L)	47.24^a^	44.26^b^	45.37^b^	47.27^a^	47.53^a^	47.57^a^	1.273	0.028
Propionate (mmol/L)	17.49	17.11	17.07	17.13	17.13	17.17	0.632	0.427
A/P	2.70	2.59	2.66	2.87	2.77	2.77	0.113	0.143
Butyrate (mmol/L)	12.77^a^	11.28^b^	12.10^a^	12.75^a^	12.17^a^	12.24^a^	0.579	0.036
Ammonia-N (mg/100 mL)	12.49	12.84	12.23	12.77	12.86	12.52	0.981	0.706

CON, control treatment; SCK, subclinical ketosis. SCK = subclinical ketosis with thiamine supplement. SEM, standard error of the mean. “a,b” in the same row means a significant discrepancy between all treatments.

### Metabolomic responses to thiamine supplementation in subclinical ketosis dairy cows

**Figure 1:**
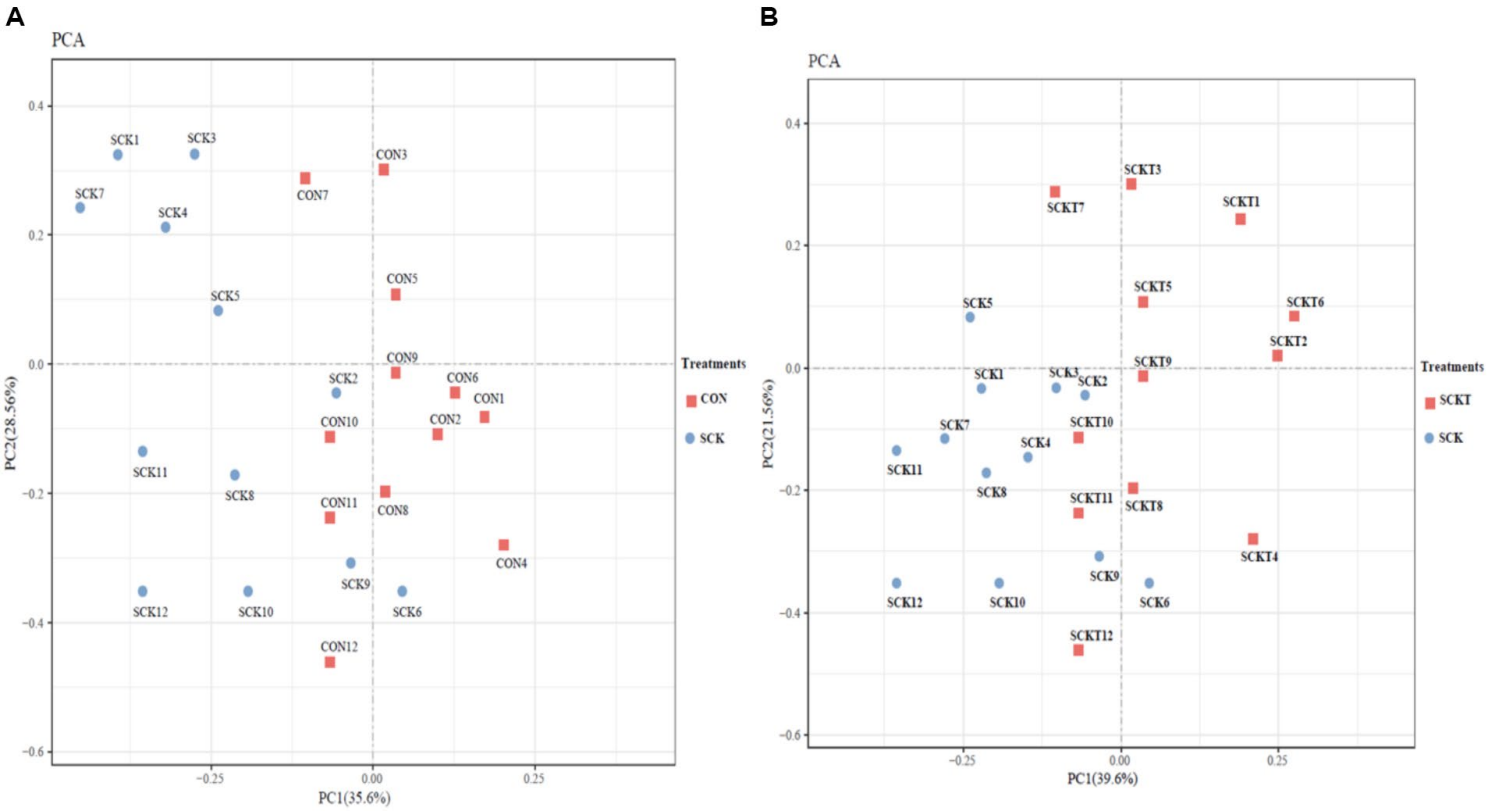
(**A)** Principal component analysis (PCA) on the whole metabolites of rumen between CON and SCK treatments. (**B)** Principal component analysis (PCA) on the whole metabolites of rumen between SCK and SCKT treatments.

Rumen metabolomic responses to thiamine supplement were determined, and the results are listed as follows. A total of 831 metabolites were identified based on the LC–MS measurement method, and all results are listed in additional Table 1. All identified metabolites were first applied to PCA analysis to determine discrepancies between SCK and CON cows, and between SCKT and SCK cows. The results are shown in Figure 1. Figure 1A shows the differential analysis between SCK and CON cows; PC1 and PC2 attributed to 35.6 and 28.56% of the total variation, respectively. Metabolites from SCK cows were significantly separated from those of CON cows, except CON7, SCK2, and SCK9. Figure 1B displays the PCA analysis between SCKT and SCK treatments. PC1 and PC2 accounted for 39.6 and 21.56% of the total variation, respectively. Thiamine supplementation treatment showed significant alterations in ruminal metabolites compared with SCK cows, except for SCKT7, SCK6, and SCK9.

Specifically, a total of 40 significantly differential metabolites, including 19 upregulated and 21 downregulated metabolites after thiamine supplementation, were identified based on the filtration standard of *p* < 0.05, fold change>2, and VIP > 1, and all these results are shown in Table 5. As shown in Table 5, the upregulated metabolites after thiamine treatment mainly include organic acids, FMN, and isobutyl acetate. The downregulated metabolites mainly include biogenic amines, amino acid derivatives, and xanthine.

**Table 5:** Significant differential rumen metabolites after thiamine supplement (*n* = 12).

Items		log2FC	*p*-value	VIP
Up regulated metabolites after thiamine supplement cows	2-Dehydro-3-deoxy-D-gluconate	1.03	0.049	1.26
	D-Leucic acid	1.05	0.048	1.17
	Ethyl vanillate	1.07	0.023	1.37
	Galactonic acid	1.07	0.036	1.29
	Uric acid	1.07	0.017	1.33
	stearic acid	1.11	0.017	1.45
	L-Glutamine	1.12	0.004	1.75
	Homovanillic acid	1.2	0.026	1.37
	2-Pyrocatechuic acid	1.24	0.031	1.11
	Isobutyl acetate	1.33	0.026	1.37
	Quercetin 3-O-glucoside	1.35	0.036	1.07
	Hippuric acid	1.42	0.017	1.23
	2,5-Dihydroxybenzoic acid	1.44	0.017	1.50
	Histidine-Tyrosine	1.58	0.097	1.13
	4-Vinylphenol	1.58	0.096	1.12
	Streptomycin	1.6	0.026	1.36
	FMN	2.85	0.040	1.36
	Salicin	2.97	0.021	1.37
	5-Dehydro-4-deoxy-D-glucuronate	3.01	0.016	1.48
Down regulated metabolites after thiamine supplement cows	Terpineol	−3.47	0.011	1.56
	Arginyl-Cysteine	−3.4	0.031	1.43
	(2S,3S,4R)-2-aminohexadecane-1,3,4-triol	−3.24	0.003	1.63
	2′-deoxyguanosine monohydrate	−3.9	0.012	1.62
	Hypoxanthine	−3.86	0.001	1.91
	Digoxigenin	−3.85	0.016	1.57
	Xanthine	−3.81	0.004	1.83
	2,6-dihydroxypurine	−3.12	0.003	1.88
	2′-O-methyluridine	−2.96	0.009	1.75
	5′-deoxyuridine	−2.94	0.042	1.29
	4-deoxypyridoxine 5′-phosphate	−2.66	0.006	1.85
	3-dehydrosphinganine	−2.48	0.008	1.66
	Deoxyinosine	−2.25	0.020	1.40
	N-acetyl glucosaminyl asparagine	−2.04	0.013	1.53
	Prolyl-asparagine	−2.02	0.008	1.74
	Asparaginyl-Asparagine	−1.99	0.022	1.46
	Rhapontigenin	−1.96	0.017	1.69
	Deoxyguanosine	−1.9	0.037	1.41
	Glycerophosphocholine	−1.75	0.013	1.76
	3-Methyl-2-oxovaleric acid	−3.02	0.019	1.54
	D-Urobilinogen	−3.21	0.009	1.76

**Figure 2:**
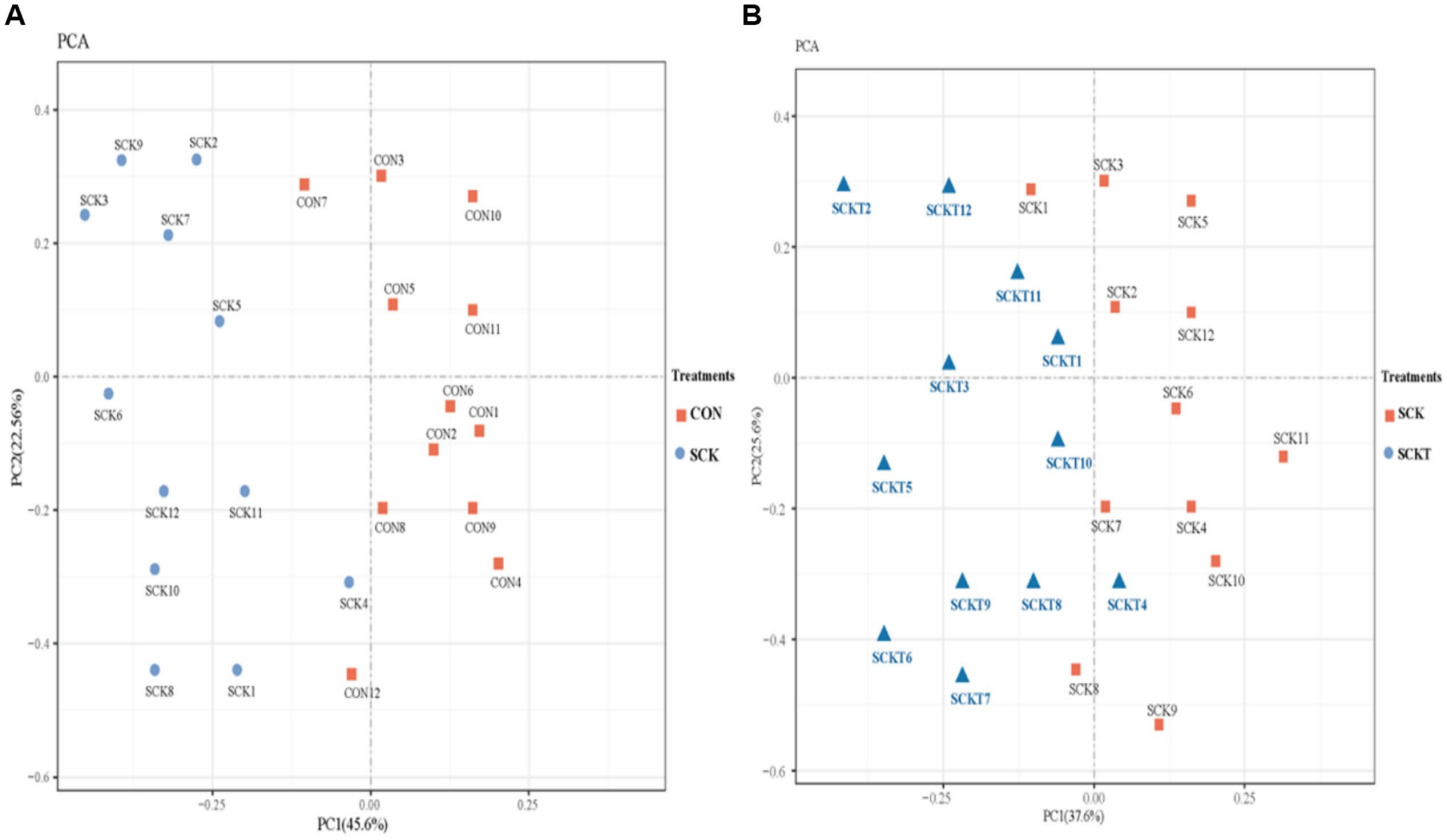
**(A)** Principal component analysis (PCA) on the whole metabolites of blood between CON and SCK treatments. (**B)** Principal component analysis (PCA) on the whole metabolites of blood between SCK and SCKT treatments.

Blood metabolomic responses to thiamine supplementation were determined, and these results are listed as follows. A total of 679 metabolites were identified using LC–MS, and all results are listed in additional Table 2. All identified metabolites were first applied to PCA analysis to determine the discrepancies between SCK and CON cows and SCKT and SCK cows. The results are shown in Figure 2. Figure 2A shows the differential analysis between SCK and CON cows, PC1 and PC2 attributed to 45.6 and 22.56% of the total variation, respectively. Metabolites from SCK cows were significantly separated from those of CON cows, except CON7. Figure 2B displays the differential analysis between SCKT and SCK treatments. PC1 and PC2 accounted for 37.6 and 25.6% of the total variation, respectively. Thiamine supplementation treatment showed a significant alteration in blood metabolites compared with SCK cows, except SCK1.

Specifically, a total of 32 significantly altered metabolites, including 15 downregulated and 17 upregulated metabolites, were identified in thiamine-supplemented treatment compared with SCK. All significantly altered metabolites are listed in Table 6. Similar to the differential metabolites in the rumen, the downregulated metabolites mainly include biogenic amines, ginkgolide B, and 2-nonanone, while the upregulated metabolites after thiamine treatment mainly include organic acids, tris-acetate, pantothenate, fluoroacetate, and (N(omega)-L-arginino) succinic acid.

**Table 6:** Significant differential blood metabolites after thiamine supplement (*n* = 12).

Items		log2FC	*p*-value	VIP
Down regulated metabolites after thiamine supplement cows	Ginkgolide B	−2.26	0.006	1.74
	2-phenylacetamide	−2.23	0.025	1.48
	5′-deoxyuridine	−2.20	0.038	1.25
	N-Acetyl-L-phenylalanine	−2.18	0.084	1.04
	Guanosine	−2.12	0.017	1.62
	Deoxyinosine	−2.09	0.056	1.15
	Serinamide	−2.04	0.097	1.13
	Spermidine	−2.02	0.096	1.12
	Tetradecanoylcarnitine	−1.86	0.054	1.14
	coformycin	−1.84	0.082	1.04
	Tetrahydrodipicolinate	−1.84	0.064	1.10
	Deoxyadenosine	−1.84	0.082	1.17
	2-nonanone	−1.81	0.029	1.30
	Gabaculine	−1.76	0.087	1.15
	Fructoselysine	−2.15	0.042	1.40
Up regulated metabolites after thiamine supplement cows	Galactonic acid	1.25	0.003	1.88
	Uric acid	1.26	0.004	1.60
	Stearic acid	1.28	0.002	1.67
	L-Glutamine	1.29	0.002	1.95
	Glutaric acid	1.58	0.008	1.74
	Harmaline	1.30	0.032	1.42
	tris-acetate	1.31	0.044	1.23
	Citicoline	1.32	0.001	1.92
	Nicotinamide riboside	1.32	0.012	1.71
	Dodecanedioic acid	2.38	0.035	1.60
	Suberic acid	2.38	0.001	1.82
	Folic acid	2.41	0.036	1.35
	Citronellyl acetate	2.74	0.003	1.84
	(N(omega)-L-arginino)succinic acid	1.86	0.044	1.49
	Kynurenic acid	1.86	0.040	1.42
	Pantothenate	1.88	0.000	2.05
	Fluoroacetate	1.89	0.027	1.51

### Functional analysis of significantly altered metabolites

**Figure 3:**
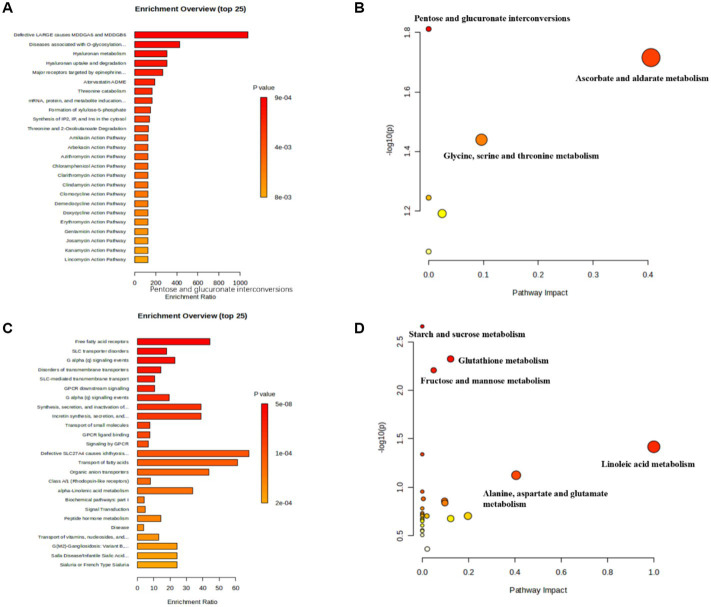
Enrichment and pathway analysis of ruminal significantly differential metabolites between SCKT and SCK treatments. **(A)** Enrichment analysis on significantly downregulated metabolites in SCKT dairy cows. **(B)** Pathway analysis on significantly downregulated metabolites in SCKT dairy cows. **(C)** Enrichment analysis on significantly upregulated metabolites in SCKT dairy cows. **(D)** Pathway analysis on significantly upregulated metabolites in SCKT dairy cows.

Functions included in the enrichment and pathway analysis of rumen differential metabolites between SCKT and SCK cows are listed in Figure 3. Figures 3A,B show the enrichment and pathway results for the downregulated metabolites in SCKT dairy cows. Diseases associated with O-glycosylation, hyaluronan metabolism, and threonine catabolism are the mainly enriched functions, while pentose and glucuronate interconversions; glycine, serine, and threonine metabolism; and ascorbate and aldarate metabolism are the mainly enriched pathways of the downregulated metabolites of SCKT dairy cows. Figures 3C,D show the enrichment and pathway results of the upregulated metabolites in SCKT dairy cows. The significantly downregulated metabolites are mainly enriched for processes related to free fatty acid receptors, SLC transporter disorders, and disorders of transmembrane transporters, and for pathways such as starch and sucrose metabolism, glutathione metabolism, fructose and mannose metabolism, linoleic acid metabolism, and alanine, aspartate, and glutamate metabolism.

**Figure 4:**
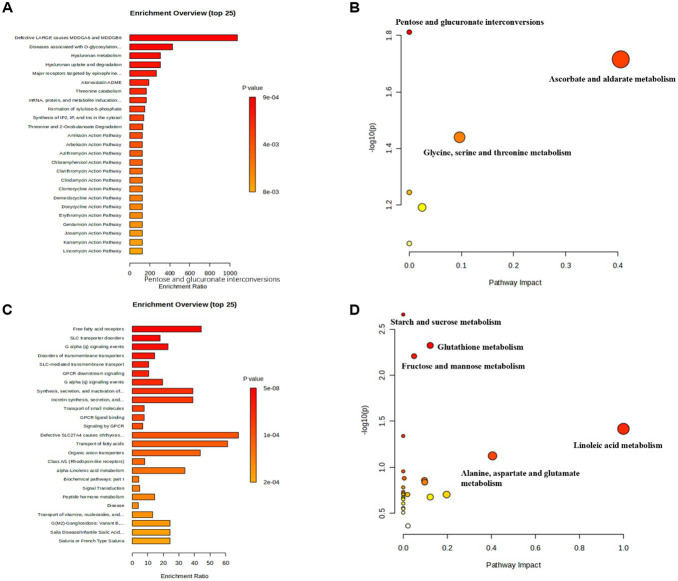
Enrichment and pathway analysis on significantly differential blood metabolites between SCK and SCKT treatments. **(A)** Enrichment analysis on the significantly upregulated metabolites in SCKT dairy cows. **(B)** Pathway analysis on the significantly upregulated metabolites in SCKT dairy cows. **(C)** Enrichment analysis on the significantly downregulated metabolites in SCKT dairy cows. **(D)** Pathway analysis on the significantly downregulated metabolites in SCKT dairy cows.

The same functional analysis of blood differential metabolites between SCKT and SCK treatments in dairy cows is listed in Figure 4. Figures 4A,B show the enrichment and pathway analysis of the upregulated metabolites of thiamine supplement-treated dairy cows. Biochemical pathways I and adenine phosphoribosyl transferase are the most enriched functions, while pyruvate metabolism, glycolysis or gluconeogenesis, tyrosine metabolism, glycerophospholipid metabolism, and phenylalanine, tyrosine, and tryptophan biosynthesis pathways are the most enriched pathways of the upregulated metabolites of SCKT dairy cows when compared with SCK. Figures 4C,D show the enrichment and pathway results of the downregulated metabolites in SCKT dairy cows. Significantly downregulated metabolites are mainly enriched in the flavan-3-ol metabolism pathway, amino acid conjugation, and carboxylic acid conjugation, as well as the pathways of ascorbate and aldarate metabolism, glycerophospholipid metabolism, pentose and glucuronate interconversions, and riboflavin metabolism.

## Discussion

### Modulatory effects of thiamine on BHBA metabolism

Subclinical ketosis in dairy cows is typically induced by the accumulation of *β*-hydroxybutyric acid (BHBA), which is synthesized in the liver from a byproduct of incomplete lipid degradation, acetoacetyl-CoA (15). In this study, thiamine supplementation suggested that it may effectively alleviate SCK by inhibiting hepatic BHBA synthesis and promoting extrahepatic BHBA utilization.

Specifically, thiamine functionally enhances the conversion of acetoacetyl-CoA to acetyl-CoA by promoting acetyl-CoA utilization as a cofactor for TPP, which effectively reduces the substrate availability for BHBA synthesis (16, 17). Additionally, elevated hepatic thiamine levels stimulate the conversion of *α*-ketoglutarate to succinyl-CoA via *α*-ketoglutarate dehydrogenase, which increases the hepatic succinyl-CoA pool. Elevated succinyl-CoA further inhibits the activity of 3-hydroxy-3-methylglutaryl-CoA synthase (HMGCS)—a key enzyme involved in BHBA synthesis (8), and subsequently leads to a significant reduction in hepatic BHBA production.

In extrahepatic tissues, thiamine enhances BHBA utilization by increasing succinyl-CoA availability. Increased circulating thiamine enters peripheral tissues and upregulates *α*-ketoglutarate dehydrogenase activity, which boosts succinyl-CoA production and enriches the systemic succinyl-CoA pool. Succinyl-CoA serves as an essential substrate for succinyl-CoA:3-oxoacid CoA transferase (SCOT), an enzyme that catalyzes the conversion of BHBA to acetoacetyl-CoA in peripheral tissues (9). Thus, thiamine supplementation improves peripheral BHBA catabolism, thereby further alleviating SCK.

### Modulatory effects of thiamine on physiological energy metabolism

Restoring a positive energy balance by improving the energy supply—primarily derived from carbohydrate metabolism—is a crucial strategy for alleviating SCK in dairy cows (18). Thiamine supplementation in our present study effectively alleviated SCK, mainly attributed to the following three aspects.

Primarily, thiamine supplementation significantly elevated ruminal acetate concentrations compared with SCK dairy cows, the key energy substrate for physiological activities in dairy cows, which was consistent with Dankel et al. (19). Increased acetate content might be attributed to the activation of pyruvate formate-lyase (PFL) after thiamine supplementation, which functions as a core enzyme that degrades pyruvate to acetyl-CoA with the assistance of cofactors S-adenosylmethionine and TPP. Acetyl-CoA is subsequently converted to acetate, which is then transported across the ruminal epithelial membrane to peripheral tissues to provide energy for basic metabolic processes (20). The significant elevation of ruminal acetate content and the acetate/propionate ratio in our study further confirms the energy-enhancing efficacy of thiamine supplementation. Notably, acetate also serves as a critical precursor for milk fat synthesis, which may explain the increased milk fat content observed in SCK cows after thiamine administration.

Thiamine supplementation significantly upregulated carbohydrate-catalytic pathways, including pyruvate metabolism, glycolysis, and gluconeogenesis, consistent with the results reported by Bohra et al. (21), which provided sufficient energy for physiological activities and further effectively reversed the negative energy balance (NEB) status. Specifically, pyruvate, as a pivotal intermediate metabolite, bridges glucose hydrolysis and the complete oxidation of carbohydrates, including the tricarboxylic acid (TCA) cycle and pentose phosphate pathway (PPP) (22). These downstream pathways generate substantial energy substrates to support various biological processes in dairy cows (17). In this energy-generating cascade, thiamine plays an indispensable catalytic role: it forms thiamine pyrophosphate (TPP), a coenzyme that mediates the conversion of pyruvate to acetyl-coenzyme A (acetyl-CoA)—the rate-limiting step initiating the TCA cycle (13). Our results suggest that thiamine supplementation increases the circulating thiamine concentration in SCK cows, which accelerates carbohydrate utilization and boosts energy supply, thereby exerting a therapeutic effect on SCK.

### Thiamine supplementation alleviates inflammatory responses of SCK dairy cows

Thiamine is one of the water-soluble vitamins that plays essential roles in the proper functioning of many body systems. Previous research mainly considered thiamine deficiency disorders, which may cause neurodegenerative disorders; nevertheless, the excess thiamine is excreted directly from the body through urine (23) and would not cause subsequent disorders, including inflammatory responses. Traditionally, biogenic amines and lipopolysaccharide (LPS) are key metabolic secondary metabolites accumulated during SCK progression, and induce robust inflammatory responses in dairy cows. As previous studies showed, biogenic amines generated are closely associated with ruminal dysbiosis, which significantly disrupts regular microbial protein synthesis and enhances the process of microbial decarboxylation of amino acids (9, 15). Our results of increased MCP content after thiamine supplementation indicated that thiamine supplementation may suppress the activity of microbial amino acid decarboxylases and promote regular microbial protein synthesis, thereby reducing biogenic amine production.

Furthermore, LPS generally induces inflammatory responses due to decreased ruminal bacterial communities and intensive TLR4-mediated NF-κB signaling pathway. Thiamine supplementation in the present and our previous studies showed significant inhibition of the NF-κB signaling pathway, further restraining inflammatory responses (24, 25) and lowering the negative impacts of SCK.

## Conclusion

**Figure 5:**
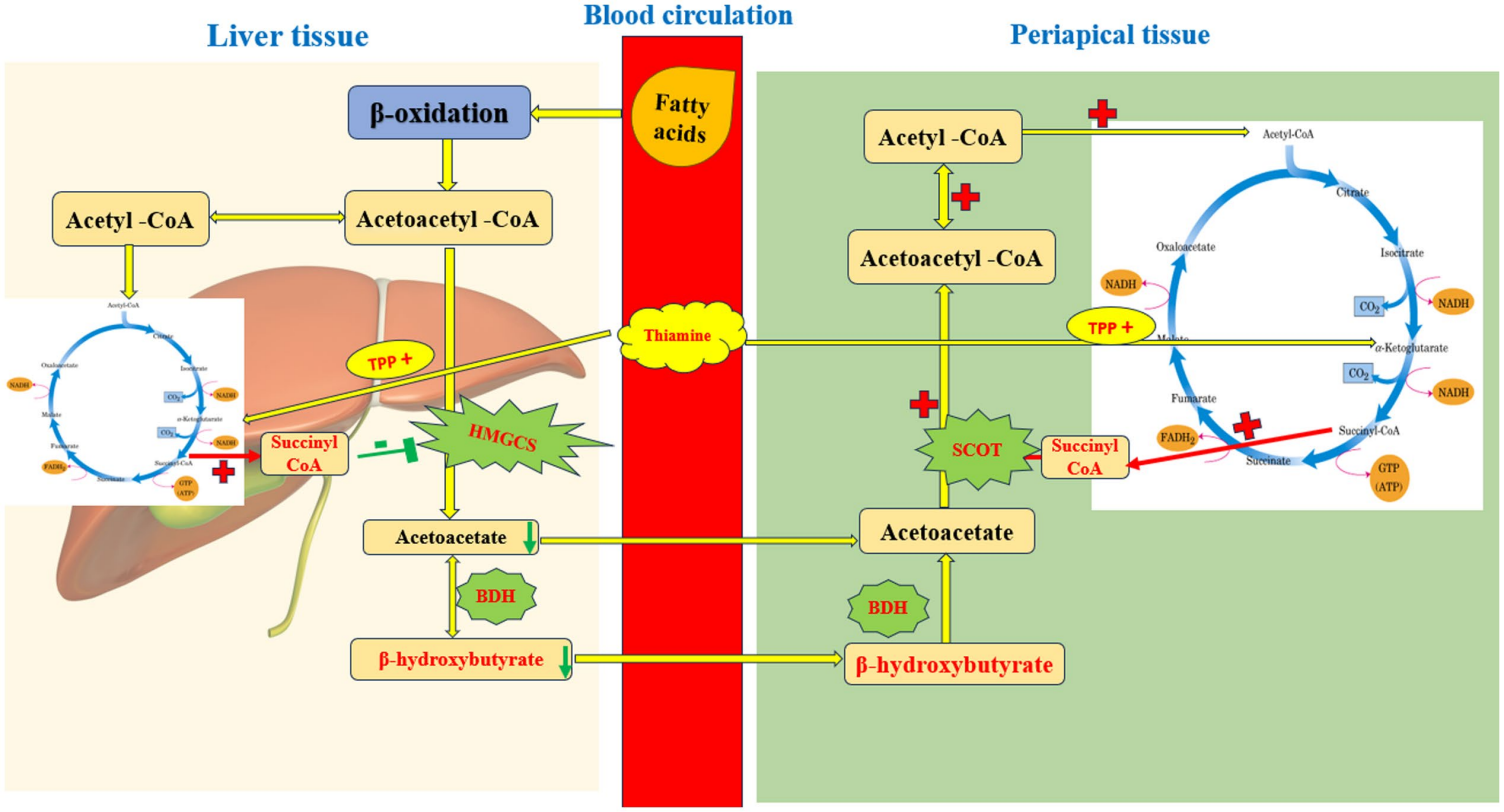
Metabolomic insights into thiamine-mediated metabolism in treating subclinical ketosis (SCK) in dairy cows.

In conclusion, the underlying pathways mediating the SCK-alleviating effects of thiamine supplementation are shown in Figure 5. Thiamine supplementation effectively mitigates subclinical ketosis in dairy cows by affecting succinyl-CoA metabolism to modulate the synthesis and catabolism of BHBA, which restores ruminal fermentability and ultimately improves milk yield and quality. These findings provide novel insights into the nutritional regulation of SCK and lay a theoretical foundation for the application of thiamine in dairy cow production.

## Data Availability

The datasets presented in this study can be found in SRC repositories. The accession number(s) can be found below: https://www.ncbi.nlm.nih.gov/, PRJNA867507.
